# Exposure to Adverse Events and Associations with Stress Levels and the Practice of Yoga: Survey Findings from a Population-Based Study of Diverse Emerging Young Adults

**DOI:** 10.1089/acm.2020.0077

**Published:** 2020-06-10

**Authors:** Dianne Neumark-Sztainer, Melanie M. Wall, Jongwoo Choi, Daheia J. Barr-Anderson, Susan Telke, Susan M. Mason

**Affiliations:** ^1^ Division of Epidemiology and Community Health, School of Public Health, University of Minnesota, Minneapolis, MN, USA.; ^2^ Department of Biostatistics, Mailman School of Public Health, Columbia University, New York City, NY, USA.; ^3^ Department of Statistics, Columbia University, New York City, NY, USA.; ^4^ School of Kinesiology, University of Minnesota, Minneapolis, MN, USA.

**Keywords:** yoga, stress, adverse events, discrimination, diversity, young adults

## Abstract

***Objectives:*** This study examines the prevalence of exposure to adverse events and associations with stress levels among a diverse population-based sample of young people. The study further explores whether these vulnerable populations, who have the potential to benefit from the mind–body practice of yoga, engage in a regular yoga practice.

***Design:*** EAT 2018 (Eating and Activity over Time) is a population-based study in which survey data were collected from 1568 ethnically/racially diverse (81.2% nonwhite) emerging young adults (mean age: 22.0 ± 2.0 years).

***Results:*** Exposure to adverse events was highly prevalent. For example, 43.9% reported at least one adverse childhood experience (ACE) (e.g., physical, emotional, or sexual abuse before age 18), whereas 40.1% reported experiencing discrimination. Exposure to adverse events was associated with higher stress levels. Practicing yoga at least 30 min/week was reported by 12.7% of the population, with variation across sociodemographic characteristics. Young adults exposed to adverse events were either more or similarly likely to practice yoga than young adults not reporting adverse events.

***Conclusions:*** The high prevalence of exposure to adverse events and associations with higher levels of stress points to a need for public health interventions. Thus, it was promising to find that young people exposed to adverse events, who may have greater emotional burdens, practice yoga at equal or greater proportions to those without these exposures. Given the potential benefits of yoga for populations living with high stress, it is important to develop further outreach efforts and provide accessible, acceptable, and affordable opportunities for practicing yoga.

## Introduction

Adverse experiences such as childhood abuse, victimization, discrimination, and financial stress are highly prevalent social determinants of health. Individuals with histories of childhood abuse are at greater risk for suicide attempts,^1^ substance use,^1^ higher weight status,^2^ and cardiovascular disease.^1,3^ Racial discrimination and other stressful life events have likewise been linked to poor health outcomes, including mental health disorders and cardiovascular disease.^4–7^ A key mechanism by which adversities across the life course may influence health is through heightened stress, which can provoke both behavioral adaptations, such as use of drugs and alcohol to soothe distress, and cause direct physiologic maladaptation through wear and tear of organ systems involved in the stress response.^8,9^ Given the potential long-term impacts of exposure to adverse experiences, it is critical to ensure that individuals exposed to these experiences can access strategies for modulating their stress levels.

Yoga is a mind–body practice that involves physical postures, breathwork, mindfulness, and meditation.^10–14^ Through linking meditative breath to movement, yoga may lead to calming of the mind, decreased reactivity to challenging situations, and better stress management.^15–17^ Several scientific reviews conclude that yoga may have benefits in terms of reducing stress.^18–21^ Thus, the practice of yoga may be helpful for populations exposed to adverse events if they have opportunities for engaging in yoga and choose to do so. Yoga is becoming increasingly popular in the United States.^1,22–24^ Practicing yoga over the past year was reported by 9.5% of adults in 2012 and increased to 14.3% in 2017.^25^

Despite the growing popularity of yoga, it is not clear if the practice is reaching those who might most benefit from it. The practice of yoga, with its potential to reduce stress and improve overall health, may be most needed by populations facing challenging situations, such as exposure to adverse life events. However, populations with limited financial resources may be most burdened by adverse life events, and some studies suggest that low-income populations are also less likely to practice yoga than those at higher income levels.^22,26^ However, the authors are unaware of any population-based studies that have examined the prevalence of practicing yoga among ethnically and socioeconomically diverse young adults who have been exposed to adverse life events.

This study examines the prevalence of exposure to adverse events in a diverse population of young people and associations with reported levels of stress. This study further examines whether young people from diverse backgrounds, and those exposed to adverse events are engaging in the practice of yoga. Specific research questions to be addressed are as follows: (1) What is the prevalence of exposure to adverse events, including adverse childhood experiences (ACEs), other stressful life events, discrimination, and financial struggles in a diverse population-based sample of adolescents and emerging young adults? (2) Do young people exposed to these adverse events have higher stress levels? (3) What is the prevalence of practicing yoga in young people across sociodemographic characteristics and among those exposed to adverse and stressful experiences? (4) How is yoga cross-sectionally related to stress levels in the overall population and in those exposed to adverse events? Findings have implications for improving the health of vulnerable populations in general, and more specifically, can inform outreach and teaching within community-based settings in which yoga is taught.

## Materials and Methods

### Study design and population

EAT 2018 (Eating and Activity over Time) is an observational epidemiologic study examining eating, activity, and weight-related health and associated factors, in a population-based sample of young people. EAT 2018 is a follow-up survey to an earlier baseline study conducted in the academic year 2009–2010 (EAT 2010). At baseline participants were middle school and senior high school students at 20 urban public schools in Minneapolis-St. Paul, Minnesota who completed classroom surveys and anthropometric measures.^27–29^ EAT 2018 followed up 8 years later through online or mailed paper surveys. This study includes cross-sectional survey data collected in the year 2017–2018 from 1568 emerging young adults (age range: 18–26; mean age = 22.0 ± 2.0 years). The University of Minnesota's Institutional Review Board Human Subjects Committee approved all protocols.

The 1568 young people who completed surveys at both assessments represents 65.8% of the original participants for whom current contact information was available at EAT 2018. Inverse probability weighting (IPW) was used for all analyses to account for missing data.^30,31^ IPW minimizes potential response bias due to missing data and allows for extrapolation back to the original EAT 2010 school-based sample. Weights for IPW were derived as the inverse of the estimated probability that an individual responded at the two time points based on characteristics reported in 2010, including demographics, past year frequency of dieting, and weight status. Demographic characteristics of the weighted sample included in the current analysis are shown in Table 1.

**Table 1.** Sociodemographic Characteristics of Study Population (*N* = 1568; Weighted Percentages)

	n	%
Age (years), mean (SD)		
Age categories	22.1 (2.0)	
18–21	712	42.7
22–25	802	53.1
26–30	54	4.1
Gender		
Male	649	46.3
Female	908	53.1
Different identity	11	0.6
Ethnicity/race		
White	366	18.8
Black	345	29.0
Hispanic	274	16.9
Asian	355	19.8
Native American	63	3.7
Other/mixed	165	11.8
Socioeconomic status		
Low	565	39.4
Low-middle	334	22.2
Middle	257	17.9
High-middle	241	13.1
High	134	7.5

### Survey development and variables

Key items from the EAT 2010 survey were included on the follow-up EAT 2018 survey.^3,27,32^ Additions to the survey were also made to assess areas of emerging interest and to reflect participants' transition from adolescence to young adulthood. To inform survey development, three focus groups (*n* = 29) were conducted in which participants first completed the EAT 2018 survey and were asked to provide input on survey content and data collection strategies. The final survey was then fielded and test–retest reliability of measures was examined using data from a subgroup of 112 participants who were asked to complete the EAT 2018 survey twice within a period of 3 weeks.

Variables used in this study include exposure to adverse events in childhood (ACEs), over the lifetime (stressful life events), or currently occurring (discrimination, and current financial struggles); past-month stress level, yoga practice, and sociodemographic characteristics. Variables, including survey questions, psychometric properties, and sources, are described in Table 2.

**Table 2.** Variables Assessed in Study and Description of Survey Items

Variables	Description of survey items
ACEs	Adverse experiences in childhood (<18 years) were assessed by asking participants about their own experiences of physical, emotional, and sexual abuse as well as three questions regarding other dimensions of dysfunction in their childhood household. The authors defined participants as exposed to each type of maltreatment using the following definitions, informed by the ACEs Scale.^49,50^*Physical abuse:* ever being hit by a family member so hard that it left bruises or marks. *Emotional abuse:* an adult in the family saying hurtful or insulting things “often” or “very often.” *Sexual abuse:* ever experienced unwanted sexual touching or forced sex from an adult in one's family and/or from an adult outside one's family. Participants were also asked to answer three questions (yes/no) regarding whether a household member was “a problem drinker or alcoholic, used street drugs, or abused prescription drugs”; was “depressed, mentally ill, or attempted suicide”; or “went to prison.” An indicator variable was defined to represent none or at least one adverse experience (test–retest agreement = 85%).^51^
Stressful life events	Participants indicated whether or not they had experienced the following six events: “had problems with the police”; “been hit, shoved, held down or had some other physical force used against you by a spouse or someone you were dating”; “been forced to touch a dating partner or spouse sexually or had some type of sexual behavior forced on you”; “been attacked, beaten, or mugged (not including events that involved a parent, caretaker, teacher, spouse, or dating partner)”; “had a close family member or friend die violently”; and “witnessed a situation in which someone was seriously injured or killed, or in which you feared someone would be seriously injured or killed.” Survey items were based on the Life Events Questionnaire,^2^ Brief Trauma Questionnaire,^52^ and previous Project EAT surveys.^53^ A dichotomous variable was created to represent having experienced any of the six events at any point (test–retest agreement = 85%).
Discrimination	Participants were asked how often they experience three forms of interpersonal discrimination: (1) “You are treated with less respect or courtesy than other people,” (2) “You receive poorer service than other people in restaurants and stores,” and (3) “People act as if they think you are not smart or clever.” Items were selected from tools that have been used to assess the impact of stigma and discrimination in longitudinal studies.^54^ Response options for each item were “Never,” “Less than once a year,” “A few times a year,” “A few times a month,” and “At least once a week.” Frequency responses for the three forms of discrimination were summed with a range of scores from 3 to 15 (Cronbach's α = 0.83, test–retest *r* = 0.69) and then dichotomized at 7 corresponding to these three types of discrimination happening on average at least a few times a year.
Financial situation	“How difficult is it for you to get by financially right now?”^55–58^ Response options included “Not at all difficult,” “Somewhat difficult,” “Very difficult or can barely get by,” and “Extremely difficult or impossible” (test–retest *r* = 0.72). Responses were dichotomized such that only responses indicating very difficult or extremely difficult challenges were taken to represent financial struggles (test–retest agreement = 92%).
Yoga practice	Young adults indicated if they had ever done yoga over the past year (yes/no) (test–retest agreement = 89%). Those who had ever done yoga were additionally asked, “On average, how frequently did you do yoga over the past year?” Seven response options ranged from “less than ½ hour/week” to “10+ hours/week.” Respondents who engaged in yoga at least 30 min/week were identified as *practicing yoga* (test–retest agreement = 86%).
Perceived stress	“On a scale from one to ten, with one being not stressed at all and ten being very stressed, how would you rate your average level of stress in the past 30 days?” (test–retest *r* = 0.69). This measure of stress was previously developed for a similar population of young adults.^59^
Sociodemographic characteristics	Sociodemographic variables included age (based on date of birth), ethnicity/race, SES, and gender. Ethnicity/race was assessed at baseline with the question: “Do you think of yourself as…? (1) White, (2) Black or African American, (3) Hispanic or Latino, (4) Asian American, (5) Native Hawaiian or Pacific Islander, (6) American Indian or Native American, or (7) Other” (test–retest agreement = 98%–100%). Since very few participants reported “Hawaiian or Pacific Islander,” or did not report their ethnicity/race, they were coded as “mixed/other.” Categorization of SES was determined at baseline and was primarily based on the highest education level of either parent with adjustments made for student eligibility for free/reduced-price school meals, family public assistance receipt, and parent employment status.^60,61^ On the EAT 2018 survey, participants were given three options for gender: male, female, or different identity (please specify). There were *n* = 11 (0.6%) who responded “Different identity” and *n* = 15 (0.9%) who did not respond. Nonresponders were recoded with their gender identity (male or female) from EAT 2010. All others were coded with their gender as reported in EAT 2018.

All items were assessed on the EAT 2018 survey unless indicated otherwise.

ACE, adverse childhood experience.

### Statistical analysis

Descriptive proportions of each adverse event are reported across the whole sample. Separate linear regressions were used to estimate the association between each dichotomous adverse event as the predictor of stress, yielding mean stress levels for those exposed and not exposed to each experience. Both crude (unadjusted) models and models controlling for age, gender, ethnicity/race, and socioeconomic status (SES) (adjusted models) were run. The proportion of individuals practicing yoga across different sociodemographic groups and by the presence of each adverse event was examined by regressing yoga practice (yes/no) on each adverse event and sociodemographic characteristics in separate logistic models. Regression-adjusted proportions of individuals practicing yoga by each adverse event were obtained by back-transforming the logit at the conditional mean of the covariates (age, gender, ethnicity/race, and SES). Finally, the authors examined associations between yoga and stress. The sample size differed slightly across analyses due to intermittent missingness of items. Item missingness ranged from 1.0% to 3.9%. As previously described, to adjust for attrition, all regression analyses and percentages were weighted with the nonresponse weights (IPW) while raw sample size values are presented. All analyses were conducted in SAS software (version 9.4, 2013; SAS, Inc., Cary, NC).

## Results

### Prevalence of exposure to adverse events

At least one ACE (physical, emotional, sexual abuse, or household dysfunction before the age of 18) was reported by 45.1% (*n* = 696) of the study participants. One-third (33.2%; *n* = 500) of participants reported at least one stressful life event, such as being the direct victim of violence or having a close friend or family member die violently. Experiencing discrimination at least a few times a year, such as being treated with less respect than other people, was reported by 40.1% (*n* = 614) of the participants. Finally, 23.2% (*n* = 337) reported that it was either very difficult or extremely difficult to manage financially. Correlations between the different experiences ranged from 0.17 (between ACEs and financial struggles) to 0.31 (between ACEs and stressful life events).

### Stress levels by exposure to adverse events

The overall mean stress level in the sample was 5.9 (standard deviation = 2.6; scale range: 1–10). Perceived stress levels were significantly higher among young people exposed to adverse events (i.e., ACEs, stressful life events, discrimination, or financial struggles) than among those not exposed. The effect size in standardized mean differences of stress ranged from moderate (0.43 standard deviations) for stressful life events to large (0.74 standard deviations) for financial struggles. Associations were statistically significant (*p* < 0.001 for all comparisons) in unadjusted analyses and in analyses adjusted for age, gender, ethnicity/race, and SES (Table 3).

**Table 3.** Distribution of Exposure to Adverse Events and Associated Stress Levels

			Unadjusted results			Adjusted results^a^		
	n	% Experiencing adverse event	Stress (mean level)	SE	p	Stress (mean level)	SE	p
ACEs								
Yes	696	45.1	6.8	0.09	<0.001	6.8	0.10	<0.001
No	834	54.9	5.2	0.10		5.2	0.09	
Stressful life events								
Yes	500	33.2	6.6	0.12	<0.001	6.7	0.12	<0.001
No	1031	66.8	5.5	0.09		5.6	0.09	
Discrimination								
High	614	40.1	6.8	0.10	<0.001	6.8	0.10	<0.001
Low	909	59.9	5.3	0.09		5.4	0.09	
Financial struggles								
Very or extremely difficult	337	23.2	7.3	0.13	<0.001	7.4	0.13	<0.001
somewhat or not difficult	1176	76.8	5.5	0.08		5.5	0.08	

^a^ Adjusted for age, gender, ethnicity/race, and SES.

SES, socioeconomic status.

Stressful life events explained 3.8% of the variance in perceived stress level in the unadjusted model and 8.4% of variance after adjusting for sociodemographic characteristics. Discrimination explained 7.4% of the variance in stress level in the unadjusted model and 11.7% of the variance after adjustment. ACEs explained 9.2% of the variance in perceived stress level in the unadjusted model and 13.0% of variance in stress, after adjustment for sociodemographic characteristics. Financial struggles explained 8.9% of variance in stress level in the unadjusted model and 13.8% of the variance in stress level in the adjusted model.

### Prevalence of yoga practice

Approximately one-fifth (*n* = 335; 19.9%) of participants reported ever having practiced yoga over the past year. Among those who reported ever practicing yoga (*n* = 335), the average frequency of practicing yoga each week over the past year was <30 min/week (*n* = 123; 35.7%); 30 min to <1 h/week (*n* = 68; 20.5%); 1 h to <2 h/week (*n* = 84; 25.7%); 2+ h/week (*n* = 58; 17.5%). For all additional analyses, only those practicing for an average of at least 30 min/week over the past year (*n* = 210; 12.7% of the total study population) were coded as practicing yoga.

Table 4 shows the prevalence of practicing yoga at least 30 min/week in the entire study population by sociodemographic characteristics. Yoga practice did not differ across age. Large differences were seen across gender; 7.2% of young men, 17.2% of young women, and 36.4% of those with a different gender identity practiced yoga (*p* < 0.001). Prevalences were fairly similar between white (14.6%), black (11.1%), Hispanic (12.4%), and Asian (10.6%) participants, whereas prevalences were lower among Native Americans (7.0%) and highest among those from mixed/other backgrounds (19.4%). Yoga was practiced across all levels of SES, with the highest levels among those from the highest socioeconomic level (19.2% at highest level vs. 10.7% at lowest level).

**Table 4.** Yoga Practice (At Least 30 Min/Week) by Sociodemographic Characteristics

		No. practicing yoga	Unadjusted results		Adjusted results^a^	
	Total, N		% Practicing yoga	p	% Practicing yoga	p
Age categories						
18–21	708	85	11.4		10.8	
22–25	794	116	13.5	0.380	13.4	0.197
26+	53	9	15.5		17.1	
Gender						
Female	901	158	17.2	<0.001	17.5	<0.001
Male	643	48	7.2		7.2	
Different identity	11	4	36.4		33.5	
Ethnicity/race						
White	365	56	14.6	0.043	13.2	0.133
Black	340	42	11.1		10.9	
Hispanic	271	36	12.4		12.6	
Asian	353	37	10.6		10.7	
Native American	62	4	7.0		7.6	
Other/mixed	164	35	19.4		18.5	
SES						
Low	558	64	10.7	0.038	9.3	0.003
Low-middle	332	49	14.8		14.4	
Middle	254	29	10.9		10.3	
High-middle	241	39	15.6		16.5	
High	133	26	19.2		20.0	

^a^ Adjusted for age, gender, ethnicity/race, and SES.

### Yoga practice by exposure to adverse events

Young adults exposed to adverse events were as or more likely to practice yoga than young adults not reporting adverse events (Table 5). In unadjusted analyses, yoga was practiced by 16.2% of young adults exposed to adverse childhood events as compared with 10.0% of those not exposed (*p* < 0.001), with similar findings after adjustment for sociodemographic characteristics. Likewise, 14.8% of those exposed to stressful life events practiced yoga as compared with 11.7% of those not exposed to stressful life events, although this difference was not statistically significant (*p* = 0.060). Discrimination and financial hardship were unrelated to prevalence of yoga practice.

**Table 5.** Yoga Practice by Exposure to Adverse Events

			Unadjusted results		Adjusted results^a^	
	Total, N	No. practicing yoga	% Practicing yoga	p	% Practicing yoga	p
ACEs						
Yes	693	121	16.2	<0.001	15.8	<0.001
No	828	86	10.0		9.8	
Stressful life events						
Yes	499	79	14.8	0.083	14.8	0.060
No	1023	127	11.7		11.3	
Discrimination						
High	610	90	13.9	0.314	13.5	0.325
Low	904	116	12.1		11.8	
Financial struggles						
Very or extremely difficult	336	46	13.6	0.592	12.6	0.958
Somewhat or not difficult	1169	158	12.5		12.5	

^a^ Adjusted for age, gender, ethnicity/race, and SES.

### Perceived stress by yoga practice

In the overall population, perceived stress was higher (*p* < 0.001) among young adults practicing yoga (mean = 6.6) than those not practicing yoga (mean = 5.8) in unadjusted analyses, although the effect size was small (0.29 standardized difference). In analyses adjusting for sociodemographic characteristics, perceived stress levels remained higher for those practicing yoga (*p* = 0.014), but the effect size was reduced to a 0.19 standardized mean difference. Among young adults exposed to each adverse event, perceived stress level did not differ by yoga practice in unadjusted analyses or in analyses adjusted for sociodemographic characteristics (Table 6).

**Table 6.** Stress Levels by Yoga Practice in Young People Exposed to Traumatic Situations

	Unadjusted results			Adjusted results^a^		
	Mean stress levels (SE)			Mean stress levels (SE)		
	Practice yoga	No yoga	p	Practice yoga	No yoga	p
ACEs (*n* = 693)	7.0 (0.21)	6.7 (0.10)	0.336	6.8 (0.22)	6.8 (0.10)	0.843
Stressful life events (*n* = 276)	6.8 (0.31)	6.6 (0.12)	0.433	6.7 (0.32)	6.7 (0.12)	0.916
Discrimination (*n* = 610)	7.0 (0.25)	6.7 (0.11)	0.308	6.9 (0.25)	6.8 (0.11)	0.729
Financial struggles (*n* = 336)	7.5 (0.35)	7.3 (0.14)	0.651	7.6 (0.36)	7.4 (0.14)	0.719

^a^ Adjusted for age, gender, ethnicity/race, and SES.

## Discussion

Findings indicate a high prevalence of exposure to adverse events among an urban population-based sample of diverse young people. Furthermore, young people exposed to these adverse events reported higher levels of stress than their counterparts. Findings further indicate that young people exposed to adverse events practice yoga at equal or greater proportions to other young people without these exposures. Finally, perceived stress was positively associated with yoga practice in the overall population but did not differ across yoga practice among young people exposed to adverse events. Findings suggest the importance of ensuring that yoga teachers recognize that many of the students in their classes may have been exposed to potentially traumatic situations.

The high prevalence of reported exposures to adverse events is disturbing, inexcusable, and preventable. For example, 40% of young people reported adverse childhood events and 40% reported concerning levels of discrimination. Unfortunately, the high prevalences are not unique to the current study population. The original ACEs study, conducted among Kaiser Permanente members, found that ∼50% of the sample had experienced one or more ACEs.^33^ Likewise, 60% of respondents to the Behavioral Risk Factor Surveillance survey, a nationally representative sample, reported at least one ACE.^34^ The lower 40% prevalence in the current sample may be due to the more limited number of adversities measured (e.g., parental divorce was not included).

Not surprisingly, and similar to other studies,^35^ young people exposed to each of these adverse events had significantly higher perceived stress levels than their counterparts. The effect size in standardized mean differences of stress ranged from moderate for stressful events to large for financial struggles. Furthermore, analyses indicated that exposure to adverse events explained a high percentage of variance in perceived stress levels, particularly exposure to ACEs and current financial struggles. Higher levels of perceived stress have been shown to be associated with poorer health outcomes of major public health importance.^36–38^

Yoga was practiced by a high percentage of emerging young adults in this diverse population-based sample. Yoga was more likely to be practiced by young women than young men, which is consistent with the literature.^22,25^ In this study, young people from different ethnic/racial and socioeconomic backgrounds practiced yoga at relatively similar percentages. Differences across sociodemographic characteristics were less apparent than in other studies,^22,25^ suggesting either a unique aspect of this sample, or that among younger people, yoga is becoming more available, acceptable, and/or accessible to varied groups.

Given the growing recognition of the potential benefits of yoga for physical and emotional well-being,^17,39–42^ it was promising to see that young people who reported adverse events (i.e., ACEs, discrimination, stressful life events, and financial struggles) practiced yoga at least as often as their counterparts. Close to half (44%) of the participants reported ACEs and yoga was practiced by higher percentages of young people reporting ACEs than their peers (16% vs. 10%). Importantly, these findings indicate that approximately half of the young people in this sample who practice yoga have been exposed to at least one ACE. Although further research is needed to determine if similar patterns exist in other populations, and to determine the effectiveness of yoga in helping these young people, the authors' findings suggest that populations exposed to adverse events are engaging in a practice that, if offered sensitively, has the potential to be of help through getting in touch with their bodies, reducing stress, and practicing self-compassion.

In the overall study population, young people practicing yoga reported higher levels of perceived stress than those not practicing yoga. Among young people exposed to adverse events, practicing yoga was not found to be associated with perceived stress. These findings are cross sectional, thus inferences about direction cannot be made. Given the overall tenets of yoga and existing research showing that yoga is beneficial for stress management,^18–21^ the authors do not believe that yoga is leading to increased stress. Another explanation is that yoga may increase one's awareness to internal signs of stress through the practice of coming inward and listening. Alternatively, young people living with more stress, and/or those aware of their internal stress levels, may seek out yoga as a practice to help. The lack of such difference in stress by yoga practice among those exposed to adverse events may indicate that the adverse events themselves, rather than perceived stress, are motivating yoga practice in these groups. Further research using longitudinal data and intervention designs are needed to assess the impact of practicing yoga on stress among populations exposed to adverse events.^39,43,44^

Study strengths and limitations should be considered in interpreting the findings. Study strengths include the large and diverse nature of the study population and the assessment of exposures to potentially traumatic events. Although yoga is often suggested as a strategy for helping individuals who have experienced trauma, the authors are unaware of any other population-based studies that have examined the practice of yoga among these vulnerable populations. Survey items on yoga practice and adverse events were only assessed at follow-up, and the cross-sectional nature of the data is a study limitation. Furthermore, questions were not asked about the context in which yoga was practiced (e.g., setting, type of yoga, and price). All measures were self-reported, and individuals may not perfectly recall adversities. The authors did not assess the specific time period for adversities. Self-reported stress has been found to be modestly correlated with objective stress measures,^45^ but also reflects individual variation in what is considered “normal” stress, and may not correspond directly to stress physiology.^46^ In addition, although utilizing a community-based sample is a study strength as it allows for a determination of who is practicing yoga, the mean frequency of practicing yoga tends to be low, with relatively few respondents reporting practicing an average of >2 h a week. Finally, it is important to replicate these findings in other study populations as it is possible that yoga offered within the Minneapolis/St. Paul area is different (e.g., more available, affordable, and accessible) to that offered in other areas.

In conclusion, study findings demonstrate the high level of exposure to adverse events among young people and the associated higher levels of stress. From a public health perspective, it is imperative to address the needs of these young people. It is promising that young people from diverse ethnic/racial and socioeconomic backgrounds, in addition to subsectors of the population who had been exposed to adverse events, practice yoga. Given the potential benefits of the practice for populations living with high levels of stress, it is important to develop further outreach efforts to reach these populations and provide opportunities for practicing yoga to those who are interested that are accessible and affordable. Although yoga should certainly not be offered as a substitute for needed psychologic care, it is important for yoga teachers to know that many of their students have likely experienced adverse events in their lives and to teach in a manner that is sensitive to the needs of this population.^14,47,48^
